# Surfactant Levels in Congenital Diaphragmatic Hernia

**DOI:** 10.1371/journal.pmed.0040243

**Published:** 2007-07-31

**Authors:** Marcus Davey

## Abstract

Marcus Davey discusses a new autopsy study that found that pulmonary surfactant content is not decreased in congenital diaphragmatic hernia.

Congenital diaphragmatic hernia (CDH), which occurs in one in 2,500 live births [1], is the most life-threatening cause of lung hypoplasia and is associated with high mortality, morbidity, cost, and suffering. The lungs are abnormally “stiff” in neonates with CDH [2], necessitating mechanical ventilation, often at high airway pressures, to prevent atelectasis and maximize the surface area available for gas exchange.

The cause of low lung compliance in CDH is multifactorial and may involve aberrant remodeling of lung structure and/or a primary surfactant deficiency. In the sheep model of CDH, the compliance of liquid-filled lungs is reduced [3], indicating involvement of surfactant-independent mechanisms; liquid filling of the lungs eliminates surface tension forces generated by the alveolar air-liquid interface. Indeed, increased thickness of inter-airspace walls and/or excessive collagen deposition in lung parenchyma have been reported in CDH [4]. On the other hand, a primary surfactant deficiency has been implicated as a cause of abnormal lung hysteresis (see Glossary) in neonates with CDH, although the role of surfactant deficiency in CDH remains controversial.

Pulmonary surfactant is a phospholipid–protein complex synthesized and secreted exclusively by alveolar epithelial type II (AEII) cells that lowers surface-tension forces at the alveolar air-liquid interface, thereby increasing lung compliance. Of the four surfactant-associated proteins (SP-A, -B, -C, and -D), it is the two smaller hydrophobic proteins, SP-B and SP-C, that interact with alveolar phospholipids to prevent alveolar collapse at low lung volumes. SP-A and SP-D are centrally involved in the innate immunity of the lung. Mechanical ventilation with high levels of inspired oxygen can impair surfactant synthesis and function, and should therefore be considered when evaluating studies involving human CDH.

Why should a primary surfactant deficiency in newborns with CDH be of serious concern, given the availability of prophylactic surfactant preparations that significantly improve lung compliance, pulmonary blood flow, and gas exchange? Clinically, the question of whether infants with CDH should receive exogenous surfactant therapy is important because such therapy can transiently compromise these fragile infants with minimal respiratory reserve. Moreover, if human CDH is indeed associated with a primary surfactant deficiency, we may opt for alternative treatment strategies (such as antenatal glucocorticoids or fetal lung gene therapy) to enhance endogenous surfactant production before birth.

**Linked Research Article**

This Perspective discusses the following new study published in *PLoS Medicine:*

Boucherat O, Benachi A, Chailley-Heu B, Franco-Montoya ML, Elie C, et al. (2007) Surfactant maturation is not delayed in human fetuses with diaphragmatic hernia. PLoS Med 4(7): e237. doi:10.1371/journal.pmed.0040237

In an autopsy study of human fetuses, Jacques Bourbon and colleagues report that pulmonary surfactant content is not decreased in congenital diaphragmatic hernia.

## Current Knowledge on Surfactant Status in CDH

**Surfactant phospholipids in CDH**. Data from human studies support the premise that production of phospholipids is normal in CDH. In human fetuses with CDH examined between 33 and 38 weeks, amniotic fluid lecithin to sphingomyelin (L/S) ratios and phosphatidylglycerol (PG) levels were not different from that of historical control data [5]. In the newborn period, concentrations of phosphatidylcholine (PC) and PG, and L/S ratios in bronchoalveolar lavage fluid, appear to be normal in CDH [6]. Moreover, surfactant PC synthesis and pool size do not appear to be altered by CDH, although turnover of PC is faster in CDH, possibly due to increased catabolism and/or recycling [7].

**Surfactant-associated proteins in CDH.** Of the few studies that have examined SP expression in CDH, data are available only for SP-A. Using semiquantitative immunohistochemistry, Asabe and colleagues demonstrated decreased SP-A expression in AEII cells in the lungs of infants with CDH obtained at autopsy within 5 d after birth [8]. In another immunohistochemical study, Minowa et al. demonstrated decreased alveolar SP-A expression in three infants with CDH, although the authors did not report on individual patient demographics and ventilator support [9]. The concentration of SP-A in tracheal aspirates of infants with CDH has been shown to be either unchanged [10] or reduced [11] by CDH.

## A New Study

In a new autopsy study published in *PLoS Medicine*, Jacques Bourbon and colleagues investigated surfactant content in human fetuses with CDH compared to age-matched fetuses with nonpulmonary diseases [12]. The primary purpose of their study was to determine whether the major components of surfactant, namely disaturated PC and SPs, are altered by CDH during the latter half of fetal development. A secondary aim was to evaluate lung expression of three glucocorticoid-regulated mediators involved in AEII cell maturation, namely keratinocyte growth factor, leptin, and neuregulin 1, isoform ß1.

The study found that fetal lung disaturated PC concentration and SP expression were similar in normal fetuses and those with CDH, and increased with advancing gestational age. Whereas lung keratinocyte growth factor levels decreased with age in normal fetuses, levels did not change in fetuses with CDH. Leptin and neuregulin 1, isoform ß1 were similar in normal fetuses and fetuses with CDH and increased age. Taken together, these data indicate that both surfactant content and molecular regulators of AEII cell maturation are essentially normal in human CDH.

The findings of this new study have important clinical implications. If alveolar surfactant levels are normal in the immediate newborn with CDH, prophylactic administration of surfactant may not be of benefit in treating such infants.

## Strengths and Limitations of the Study

This new study has many strengths. First, this is the only longitudinal study to compare pulmonary surfactant content in human fetuses with C DH with age-matched controls. Irrespective of gestational age, there were no significant differences between fetuses with CDH and normal fetuses, which provides convincing evidence that surfactant content is normal in CDH. Second, unlike in previous studies, the fetal lungs were not ventilated, so the results are unaffected by extraneous factors that can alter surfactant production, specifically aggressive mechanical ventilation with high levels of inspired oxygen. Moreover, none of the mothers received antenatal glucocorticoids, which would influence surfactant content. Third, the study examined expression of all four pulmonary SPs. To date there has been no information on lung levels of the SPs that are involved in lowering alveolar surface tension forces, namely SP-B and -C.

However, there are important caveats of the study. Levels of surfactant content measured from lung tissues may not reflect that present within the airspaces, particularly if surfactant secretion is abnormal in CDH. In addition, data on pulmonary disaturated phosphatidylcholine levels were reported only up to 33 wk of gestation. It is conceivable that disaturated phosphatidylcholine levels may differ between fetuses with CDH and normal fetuses closer to full term. These caveats should be considered when interpreting results from the current study. Finally, because of the small number of fetuses, the study cannot correlate the degree of pulmonary hypoplasia and surfactant content. There is a spectrum of pulmonary hypoplasia associated with CDH, and surfactant status may be different between fetuses with severe lung growth deficits and those with milder forms of the disease.

## Unanswered Research Questions

Knowledge of alveolar surfactant levels in the immediate newborn is required to determine if CDH is associated with a primary surfactant deficiency. Such information may be difficult to obtain, because institutional review boards are unlikely to approve bronchoalveolar lavage in the newborn with severe CDH. However, it may be possible to evaluate surfactant status in fetal lung fluid that is suctioned from the airways during cesarean deliveries.

**GLOSSARY**

**Alveolar epithelial type II (AEII) cells:** The alveolar epithelium is composed of two cell types. The cuboidal type II cells synthesize and secrete surfactant and are progenitor cells for the elongated type I cells through which gas exchange occurs.

**Lecithin sphingomyelin (L/S) ratio:** The phospholipids lecithin and sphingomyelin are produced by the fetal lung and are secreted into the amniotic fluid. The relative concentration of these phospholipids is used to determine the biochemical maturity of the lung.

**Lung hysteresis:** The difference in the pressure-volume curve between inspiration and expiration.

**Phosphatidylglycerol:** A surfactant phospholipid, representing 5%–13% of all phospholipids.

**Phosphatidylcholine:** The most abundant species of phospholipids in lung surfactant, representing approximately 85% of the lipids in surfactant.

## Abbreviations

AEII: alveolar epithelial type II
CDH: congenital diaphragmatic hernia
L/S: lecithin to sphingomyelin (ratio)
PC: phosphatidylcholine
PG: phosphatidylglycerol
